# Cytotoxic Activity of *Lepidium virginicum* L. Methanolic Extract on Human Colorectal Cancer Cells, Caco-2, through p53-Mediated Apoptosis

**DOI:** 10.3390/molecules29163920

**Published:** 2024-08-20

**Authors:** Renata Gallegos-Saucedo, Tonatiuh Barrios-García, Eduardo E. Valdez-Morales, Emmanuel Cabañas-García, Alma Barajas-Espinosa, Yenny Adriana Gómez-Aguirre, Raquel Guerrero-Alba

**Affiliations:** 1 Departamento de Fisiología y Farmacología, Centro de Ciencias Básicas, Universidad Autónoma de Aguascalientes, Aguascalientes 20100, Mexico; renatagalleta@gmail.com (R.G.-S.); tonatiuh.barrios@edu.uaa.mx (T.B.-G.); eduardo.valdez@edu.uaa.mx (E.E.V.-M.); 2 Consejo Nacional de Humanidades, Ciencias y Tecnologías (CONAHCyT), Ciudad de México 03940, Mexico; yenny.gomez@edu.uaa.mx; 3 Centro de Estudios Científicos y Tecnológicos No. 18, Instituto Politécnico Nacional, Blvd. del Bote 202 Cerro del Gato Ejido La Escondida, Col. Ciudad Administrativa, Zacatecas 98160, Mexico; emmanuel.cabanasg@gmail.com; 4 Escuela Superior de Huejutla, Universidad Autónoma del Estado de Hidalgo, Huejutla de Reyes, Hidalgo 43000, Mexico; alma_barajas@uaeh.edu.mx; 5 Departamento de Química, Centro de Ciencias Básicas, Universidad Autónoma de Aguascalientes, Aguascalientes 20100, Mexico

**Keywords:** Virginia pepperweed, Brassicaceae, LC-MS/MS, human carcinoma, cell line, cytotoxicity, apoptosis, bioactivity

## Abstract

Colorectal cancer (CRC) is the third most common type of cancer worldwide. Its treatment options have had a limited impact on cancer remission prognosis. Therefore, there is an ongoing need to discover novel anti-cancer agents. Medicinal plants have gained recognition as a source of anti-cancer bioactive compounds. Recently, ethanolic extract of *L. virginicum* stems ameliorated dinitrobenzene sulfonic acid (DNBS)-induced colitis by modulating the intestinal immune response. However, no scientific study has demonstrated this potential cytotoxic impact on colon cancer cells. The objective of this study was to evaluate the cytotoxic effect of the methanolic extract of *L. virginicum* (ELv) on a human colorectal adenocarcinoma cell line (Caco-2) and to identify and quantify the phenolic compounds present in ELv extracts by liquid chromatography-mass spectrometry analysis. The cytotoxic activity was assessed using cell viability assays by reduction in the compound 3-(4,5-dimethylthiazol-2-yl)-2,5-diphenyltetrazolium bromide (MTT) and lactate dehydrogenase (LDH). MTT and LDH assays revealed that the ELv decreases cell viability in the Caco-2 cell line in a concentration-dependent manner. Cell death was a result of DNA fragmentation and p53-mediated apoptosis. Eight phenolic acids and five flavonoids were identified and quantified in the stems. In conclusion, our findings demonstrate that the extract of *L. virginicum* possesses cytotoxic properties on Caco-2 cell line, suggesting that it could be a potential source of new drugs against CRC.

## 1. Introduction

Colorectal cancer (CRC) is the second leading cause of cancer-related mortality, causing 935,000 deaths in 2020. Its incidence varies notably across countries and world regions, correlating with socioeconomic development [1]. Unfortunately, nearly 20% of patients with CRC present metastases upon diagnosis, while up to 50% of patients with initially localized disease eventually develop it [2]. During the last two decades, the prognosis of metastatic CRC (mCRC) patients has improved with the advances in surgical approaches and new medical treatment regimens [3]. However, in most cases, the currently available therapies have failed to eradicate this life-threatening disease [4]. Furthermore, decreasing life quality due to adverse effects and resistance to chemotherapy remains one of the most significant challenges in the long-term management of CRC [5]. The lack of access to chemotherapy due to the treatment expense has an additional pronounced impact on low and middle-income countries [6]. Hence, the development of new, more accessible therapeutic strategies is necessary. In this context, plant extracts are an important source of bioactive antitumor compounds that are being considered as an alternative treatment [7]. 

Extracts from the Brassicaceae family contain abundant bioactive compounds directly related to a positive effect on human health, including cancer [8]. *Lepidium virginicum* L. is a herbaceous plant from North America and Mexico belonging to this family and is commonly known as pepperwort or Virginia pepperweed. This medicinal plant is traditionally used as an expectorant, a diuretic and as a treatment for diarrhea and dysentery [9]. There are few scientific studies on the pharmacological properties of *L. virginicum*. The first report describes that the isolation of benzyl glucosinolate from the crude extract of the roots of this plant exhibited an anti-amoebic activity [10]; later, another study corroborated its antiprotozoal activity [11]. Other authors found that the essential oil of *L. virginicum* contains phenylacetonitrile and α-terpineol with antifungal activity against *Colletotrichum acutatum* in tamarillo fruits [12]. In 2011, a Korean research group reported that the aqueous root extract of *L. virginicum* induced inhibition of cell proliferation by apoptosis in HCT116 human colon carcinoma cells [13]. Recently, we found that the ethanolic extract of *L. virginicum* stem ameliorated DNBS-induced colitis via modulation of the intestinal immune response, as its administration led to a significant decrease in immune cell infiltration, MPO activity, and gene expression of CXCL1, TNF-α, and IL-1β; furthermore, we identified 28 phytochemicals including aromatics, esters and hydrocarbons by gas chromatography-mass spectrophotometry (GC-MS) and the stems of *L. virginicum* have a higher phenolic and flavonoid content than the leaves and roots [14]. Phenolic compounds constitute an important group of natural products that are associated with a wide range of pharmacological properties such as anti-microbial, anti-inflammatory, anti-diabetes and anti-cancer activities [15]. The possible cytotoxic effects of these compounds, all part of the *L. virginicum* extract, on CRC cells remain to be elucidated. Therefore, the present study aimed to investigate the anti-cancer activity of the methanolic extract of *L. virginicum* (ELv) in human colorectal cancer using Caco-2 cells as a model system. 

## 2. Results

### 2.1. Liquid Chromatography-Mass Spectrometry Analysis of L. virginicum

Employing liquid chromatography-mass spectrometry (LC-MS/MS) and targeted assay with 30 authenticated standards., we detected and quantified eight phenolic acids and five flavonoids in the methanolic extract of *L. virginicum* stems (see Table 1 and Table 2). Among the identified metabolites, the most abundant flavonoid was quercetin-3-galactoside (1965.71 ng/g), followed by quercetin-3-glucoside (379.48 ng/g), kaempferol (86.22 ng/g), quercetin (52.31 ng/g), and naringenin + naringenin chalcone (4.93 ng/g). Apigenin, apigeninidin, catechin, daidzein, delphinidin, epicatechin, genistein, hesperetin, luteolin, phloretin, proanthocyanidin A2, procyanidin B2, and rutin were not detected. On the other hand, the most abundant phenolic metabolites were cinnamic acid (3466.98 ng/g) > *p*-coumaric acid > ferulic acid > vanillic acid > caffeic acid > chlorogenic acid > syringic acid > gallic acid (see Table 2). Protocatechuic acid was not detected.

Cinnamic acid and flavonol derivatives have also been identified in hypocotyls of other *Lepidium* species, such as *Lepidium meyenii* Walp. [16]. On the other hand, for *L. sativum* seeds, Yábar et al. [17] detected the presence of 12 phenolic metabolites. Among the detected metabolites, chlorogenic acid, luteolin-7-glucoside, gallic acid, ferulic acid, dihydroquercitin, and quercetin were found as the main constituents, similar to some of the metabolites we identified in *L. virginicum* stems. This evidence suggests that chlorogenic, ferulic and gallic acids, as well as quercetin and structurally related flavonoids appearing as glycosides or aglycones, are present in different *Lepidium* species.

### 2.2. The Methanolic Extract of L. virginicum Significantly Reduced the Cellular Metabolic Activity of Caco-2 but Not of Detroit 548 Cells

**Figure 1:**
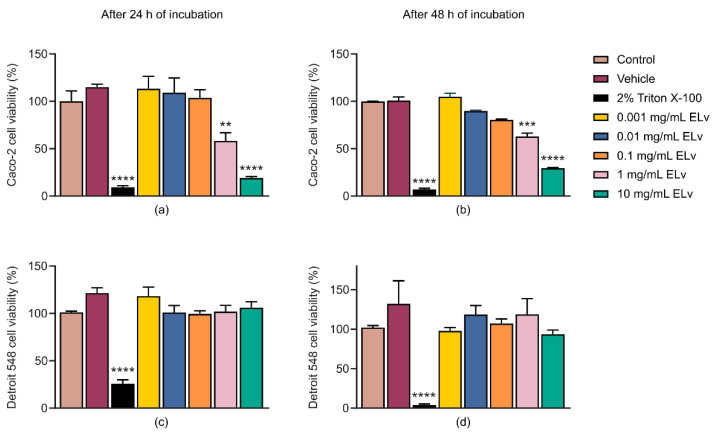
Effect of the methanolic extract of *L. virginicum* (ELv) at different concentrations on cell viability in Caco-2 cells incubated 24 (**a**) and 48 h (**b**). Effect on cell viability of Detroit 548 fibroblasts exposed to different concentrations of ELv for 24 (**c**) and 48 h (**d**). Cell viability analysis was carried out using the MTT assay. Control cells were exposed to DMEM medium, and vehicle treatment was 0.2% ethanol diluted in DMEM. The vertical lines represent the standard error (SEM) of the mean of each of the treatments. Three independent experiments were performed in triplicate. ** *p* < 0.01, *** *p* ˂ 0.001 and **** *p* ˂ 0.0001 denote statistically significant differences when comparing treatment vs. control by ANOVA test with Dunnett’s post hoc test.

Cellular metabolic activity was analyzed using 3-(4,5-dimethylthiazol-2-yl)2,5-diphenyltetrazolium bromide (MTT) assays to assess the effect of ELv on the viability of normal human fibroblast, Detroit 548, and human colon adenocarcinoma, Caco-2, cells lines after 24 and 48 h of exposure. The results showed that exposure to ELv, at concentration of 1 mg/mL for 24 and 48 h, significantly reduced cellular metabolism as a measure of Caco-2 cell viability (Figure 1a,b).

Detroit 548 cells were used to evaluate the specificity of the ELv towards cancer cells. The ELv at the different concentrations tested (0.001–10 mg/mL) had no significant changes in the cell viability of Detroit 548 cells exposed for 24 (Figure 1c) and 48 h (Figure 1d) compared to the cell viability of control cells (no treatment).

**Figure 2:**
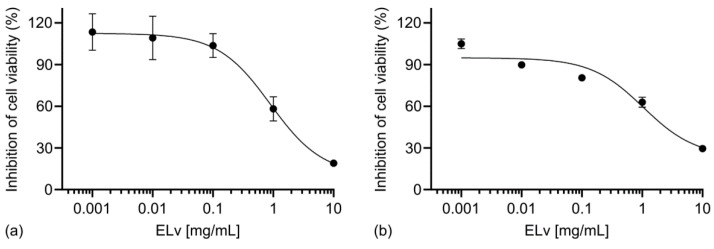
Concentration–response curves for ELv inhibitory effect (0.001–10 mg/mL) on Caco-2 cell viability for 24 (**a**) and 48 h (**b**) of incubation. Cell metabolic viability analysis was carried out using the MTT assay. Data were fitted to a three-parameter logistic function in GraphPad Prism 8 to obtain IC_50_ values. Each point represents the mean ± SEM from three independent experiments run in triplicate.

Based on the cell death rate, we calculated the half-maximal inhibitory cell viability concentration (IC50) value of ELv in Caco-2 cells at 24 h to be 0.89 ± 0.63 mg/mL (Figure 2a) and 48 h to be 1.04 ± 0.79 mg/mL (Figure 2b). 

### 2.3. ELv Increased Lactate Dehydrogenase (LDH) Release in Caco-2 and Detroit 548 Cells after 48 h of Incubation with the Highest Concentration Tested

**Figure 3:**
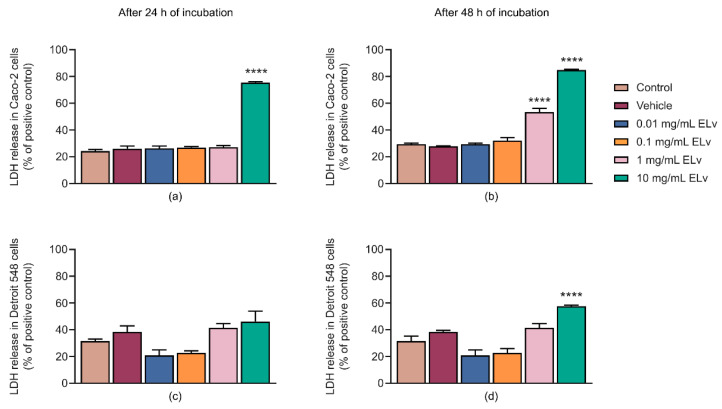
Effect of the methanolic extract of *L. virginicum* (ELv) at different concentrations on LDH released in Caco-2 cell line incubated 24 (**a**) and 48 h (**b**), as well as in Detroit 548 human fibroblast cell line in incubation periods of 24 (**c**) and 48 h (**d**). Control cells were exposed to DMEM medium, and vehicle treatment was 0.2% ethanol diluted in DMEM medium. Data are shown as the mean ± SEM of three independent experiments (*n* = 3) in triplicate. **** *p* ˂ 0.0001 denotes statistically significant differences when comparing treatment vs. control by ANOVA with Dunnett’s posthoc test.

The permeability of the cell membrane of Caco-2 and Detroit 548 cell lines after treatment with ELv at different concentrations for 24 and 48 h was determined by LDH release assay, as a measure of cytotoxic effect. Results obtained from cells incubated with 2% Triton X-100 served as a positive control and as the reference maximum (100%) response to which others were compared. After 48 h of incubation with 1 mg/mL of ELv, Caco-2 cells presented significant increases of 53% in LDH release compared to control cells (*p* ˂ 0.0001; Figure 3b), indicating that the ELv exerted a cytotoxic effect on Caco-2 cells in a time. 

On the other hand, ELv did not significantly increase the release of LDH in Detroit 548 fibroblasts post 24 h incubation with different concentrations of ELv (Figure 3c). However, prolonged (48 h) exposure to the highest tested ELv concentration proved to be highly cytotoxic (Figure 3d). Both MTT and LDH assay results indicate that the ELv-induced cell death was time- and concentration-dependent in Caco-2 cells.

### 2.4. ELv-Induced Caco-2 Cell Death via Apoptosis

**Figure 4:**
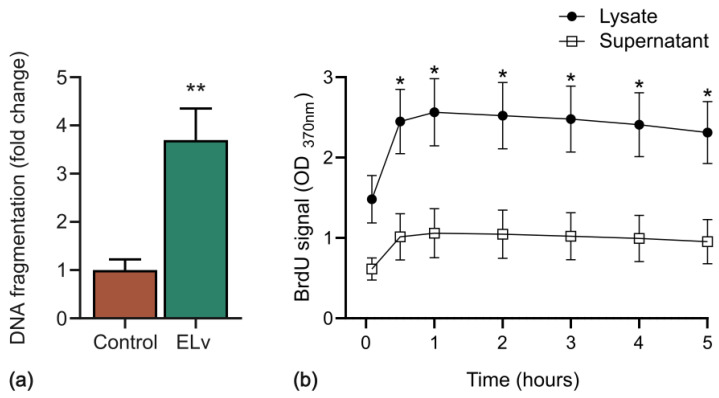
ELv-induced DNA fragmentation in Caco-2 cells. (**a**) Caco-2 cells were treated with 1 mg/mL of ELv for 48 h. The supernatant was recovered, and the fragmented DNA was quantified using the ELISA kit. Data are shown as the mean ± SEM of 4 independent experiments (*n* = 4) in triplicate. ** *p* ˂ 0.01 denotes statistically significant differences when comparing treatment vs. control by unpaired Student’s *t*-test. (**b**) Kinetics of cell death induced by ELv in Caco-2 cells. Cells labeled with BrdU were incubated with 1 mg/mL of ELv at 37°C and monitored during the first five hours of incubation. * *p* ˂ 0.05 denotes statistically significant differences when comparing treatment vs. control by two-way ANOVA with Sidak’s posthoc test.

As a high ELv concentration, 10 mg/mL, was also cytotoxic for Detroit 548 cells, we decided to use a maximum dose of 1 mg/mL ELv for the following experiments, as this was not toxic for non-cancerous Detroit 548 cells. Caco-2 cells were incubated with 1 mg/mL of ELv for 48 h, and DNA fragmentation was determined by ELISA to test whether ELv induced cytotoxicity by apoptosis. Figure 4a depicts the significant increase in DNA fragmentation of Caco-2 cells exposed to 1 mg/mL of the extract. The absorbance of BrdU-labeled DNA fragments was significantly higher, approximately 1.5-fold, in the cell cytoplasm (lysate) than in the culture supernatant within the first 0.5 h of exposure to ELv, indicating that the primary mechanism of cell death is apoptosis, although necrosis cannot be ruled out either because there also was a slight increase in DNA fragments in the supernatants at the same time of exposure to ELv (Figure 4b; *p* ≤ 0.05).

### 2.5. Real-Time PCR Analysis of Expression of Genes That Regulate Apoptosis

**Figure 5:**
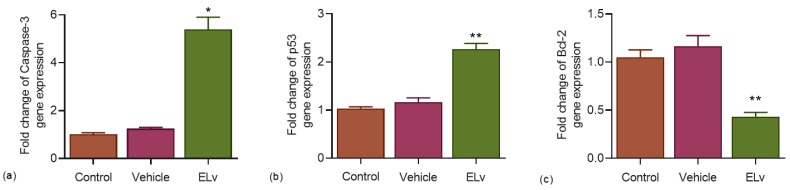
Effect of the methanolic extract of *L. virginicum* (ELv; 1 mg/mL) on the gene expression of caspase-3 (**a**), p53 (**b**) and Bcl-2 (**c**) in Caco-2 cells. Three independent experiments (*n* = 3) were performed in triplicate. Data are expressed as the mean ± S.E.M.* *p*< 0.05 and ** *p* < 0.01, which denotes statistically significant differences when comparing treatment vs. control by ANOVA with Dunnett’s test.

We analyzed the expression of three representative apoptotic genes in control versus ELv-treated Caco-2 cells to determine whether 1 mg/mL ELv altered the transcript level of apoptotic genes. Results demonstrate that ELv significantly increased the pro-apoptotic caspase-3 expression approximately 5.4-fold in treated cells compared to controls (*p* < 0.05; Figure 5a). Significant pro-apoptotic p53 gene expression was also induced with ELv. Figure 5b shows that ELv promoted a 2.2-fold increase in p53 mRNA in treated Caco-2 cells compared to controls (*p* < 0.01). On the other hand, the transcript of anti-apoptotic protein Bcl-2 was downregulated in ELv-treated cells compared to controls (Figure 5c). These results suggest that ELv-induces apoptosis in Caco-2 cells via a caspase-3 and p53-dependent mechanism. 

## 3. Discussion

CRC is the second leading cause of cancer-related death worldwide. About 20% of CRC patients are diagnosed with stage IV disease (metastatic CRC, mCRC), and approximately 14% to 50% of patients with locally advanced or early CRC go on to develop local recurrence or distant metastatic disease [2]. Different treatments are available now, such as targeted therapy and immunotherapy, and the survival outcome for CRC has increased significantly over the last two decades [18]. However, the overall survival for mCRC is averaged to be 2 to 3 years [19], associated with severe complications and high healthcare system costs [20,21]. Due to the economic burden and side effects imposed by conventional pharmaceutical treatments, the use of natural products has gained popularity, as they are recognized as an important source of anti-cancer agents. About 60% of commonly used anti-cancer drugs are obtained primarily from plants [22]. Therefore, searching for new anti-cancer products can potentially lead to novel therapeutic agents and drug targets for cancer treatment and thus improve patient survival and quality of life. 

This study demonstrated the anti-cancer properties of methanolic extract of *L. virginicum* stems. The ELv decreased the cell viability of colorectal cancer cells, Caco-2, without affecting the Detroit 548 normal human fibroblast cells after 24 h of treatment. A ten-fold decrease in ELv concentration (1mg/mL) continued to be significantly toxic for Caco-2 but not for Detroit 548 cells. A previous study using aqueous extracts of leaves and roots of *L. virginicum* also demonstrated a cytotoxic effect on HCT116 human colon carcinoma cells by inducing apoptosis [13], supporting the results of this study and suggesting that *L. virginicum*-derived anti-cancer compounds might be effective inhibitors of cancer cells. 

Our previous study found that the ethanolic extract of *L. virginicum* stems ameliorated DNBS-induced colitis via modulation of the intestinal immune response [14]. However, in the present study, we used the methanolic extract because previous reports have demonstrated that the extraction of bioactive metabolites is enhanced by using methanol [23].

Several investigations have spotlighted phenolic compounds as anti-cancer drugs. This phytochemical family contains several subclasses, such as phenolic acids and flavonoids, one of the most abundant molecules. These groups of molecules exert anti-cancer effects by acting on the multiple checkpoints of cancerous cells, inducing apoptosis, autophagy, and cell cycle arrest with high specificity [24]. For this reason, we used LC-MS/MS chromatography and certified standards to detect and quantify phenolic acids and flavonoids in the methanolic extract of *L. virginicum*. Results from LC-MS analysis revealed a more abundant presence of quercetin-3-glucoside and quercetin-3-galactoside, and, in lower concentrations: kaempferol, quercetin and naringenin all of which have been demonstrated to have cytotoxic activity [25]. Several studies have shown that quercetin plays a substantial part in suppressing cancer cells in breast, colon, prostate, ovary, endometrial, and lung tumors [26]. Furthermore, the anti-cancer effects of quercetin-3-glucoside extracted from apple pomace in human cervical cancer cells are thought to induce cell cycle arrest and apoptosis [27]. Interestingly, there appears to be a synergistic antiproliferative effect of quercetin and kaempferol in the human gut (HuTu-80 and Caco-2) and breast cancer (PMC42) cell lines [28]. Likewise, naringenin’s documented anti-cancer effect in breast cancer cells arrests cell development at the G0/G1 phase, while, in vivo, it alters the mitochondrial-mediated intrinsic pathway responsible for apoptosis [29]. This observation has also been documented in hepatocellular [30], prostate [31] and THP-1 [32] cancer cells, suggesting it as an antiproliferative compound. We also detected various phenolic acids by LC-MS, amongst which cinnamic, p-coumaric, ferulic and vanillic acids stand out and whose antiproliferative and apoptotic effects in human colon adenocarcinoma cells (HT-29) were previously demonstrated [33]. Taken together, all these studies suggest that the flavonoids and phenolic acids detected in the methanolic extract of *L. virginicum* could be participating in the cytotoxic effect observed in Caco-2 cells; however, published evidence indicates that the bioactivity of extracts depends not only on the chemical entities present in plant extracts but also on their concentration, and cancer specificity [34]. Further studies are required to assess the bioactivity of the isolated compounds and the possible synergistic effects with other metabolites. Furthermore, the possibility that other compounds not determined by LC-MS could also participate in the cytotoxic effect is not ruled out.

The results of MTT and LDH assays indicate that ELv significantly reduces the viability of Caco-2 human colorectal cancer cells in a concentration- and time-dependent manner. Surprisingly, the vehicle (0.2% ethanol) appears to increase cell viability in Detroit 548 cells in the MTT and LDH assays, suggesting a proliferative effect of ethanol in these cells. In this regard, a study found a marked difference in cellular behavior above and below 1% ethanol concentration in the mouse embryonic fibroblast cell line, NIH 3T3. They observed a two-fold increase in MTT activity at low doses of ethanol, while at high doses, it decreases. This increase in MTT activity at low doses did not imply changes in cell proliferation or mitochondrial deterioration, concluding that cells exhibit adaptation to low doses of ethanol, where recovery from ethanol-induced stress [35]. It is possible that the effect of 0.2% ethanol on the activity of the MTT and LDH assays is masking the cytotoxic effect of ELv on Detroit 548 cells and may be the reason why we observed a cytotoxic effect at the highest dose tested on Detroit cells. Therefore, additional experiments are required to test this possibility, which is outside the scope of this study. 

Cell death type induced by the ELv has been demonstrated using DNA fragmentation ELISA assay. Exposure of the ELv to Caco-2 cells led to an increase in DNA fragments in both cell lysate and culture supernatant, although the levels of the DNA fragments were significantly higher in the lysates, suggesting that the methanolic extract of *L. virginicum* could stimulate the cell death by apoptotic and necrotic mechanisms or apoptotic/secondary necrotic cell death in Caco-2 cells, as has been demonstrated in previous studies using plant extract [36]. Furthermore, we observed that treatment of the ELv elevated mRNA expression of caspase-3 and p53, confirming that this extract stimulated apoptotic cell death in Caco-2. Numerous studies have shown that various other phytochemical compounds induce cell death in different colorectal cancer cell lines through an apoptotic mechanism [37,38], supporting the results of this study. 

Caspase-3 mRNA was significantly increased in Caco-2 cells after 48 h of treatment with 1 mg/mL of ELv. Several studies have shown that caspase-3 is a crucial enzyme for apoptosis induction, essential for chromatin condensation and DNA fragmentation [39]. Activation of caspase-3 in cancerous cells has been suggested as a potential therapy for cancer treatment [40]. 

The tumor suppressor protein, p53, is a critical mediator of apoptosis and carcinogenesis [41]. In the present study, we observed a significant increase in p53 gene expression in Caco-2 cells treated with ELv compared to control cells. The p53 gene encodes a tumor suppressor protein that leads to cell growth arrest in the G1 phase and induces apoptosis after DNA damage through distinct pathways [42]. It has been reported that approximately 40–50% of sporadic CRC is related to this gene’s defect [43]. Transcript over-expression of p53 is thus likely required to execute apoptosis cancer cells. 

Two distinct pathways are associated with apoptosis: the mitochondrial, or intrinsic, pathway and the death receptor, or extrinsic, pathway. The p53 gene has been implicated in apoptotic signaling pathways [42]. The intrinsic pathway is activated by stress conditions, such as: cytokine deprivation, endoplasmatic reticulum stress, and DNA damage, and is regulated by Bcl-2 family proteins [44]. It initiates through transcriptional upregulation of BH3 pro-apoptotic protein members and inhibits prosurvival Bcl-2 proteins, thereby unleashing the cell death effector Bax and Bak, causing mitochondrial outer membrane permeabilization, with consequent activation for the caspases cascade [45]. In the present work, we found that ELv incubation reduced the Bcl-2 transcript levels in Caco-2. Previous work has demonstrated that selective silencing of Bcl-2 expression induced massive apoptosis of HCT116 colorectal cancer cells in a p53-dependent manner [46], suggesting that ELv-induced apoptosis in Caco-2 cells is p53-dependent by reducing Bcl-2 transcript. Similar results have been documented using seed extract of *Annona squamosa*, whose effects resulted in a significant down-regulation of Bcl-2 and upregulation of p53 mRNA expression in PC-3, HepG-2, Caco-2, and MCF-7 treated cell lines [47].

In conclusion, the present study demonstrates that the methanolic extract of *L. virginicum* stems has a cytotoxic effect on the Caco-2 cell line via apoptosis throughout upregulated caspase-3 and p53 expression. Although the evidence presented here suggests the therapeutic potential of *L. virginicum* extracts, further studies are required to assess the possible side effects of said extracts and the in vivo interaction of extracts with diet components and different stimuli using animal models.

## 4. Materials and Methods

### 4.1. Collection of Plant Material

The whole plant of *L. virginicum* was collected from urban fields in the city of Aguascalientes, Mexico (N: 21°54′49.8″, W: 102°18′59.5″) at an altitude of approximately 1868 m in October 2021. Biologist Julio Martínez Ramírez, from the Biology Department of the Autonomous University of Aguascalientes (UAA), identified and authenticated the plant with the specimen of *L. virginicum* previously registered with voucher No. 30775 and placed at the Autonomous University of Aguascalientes Herbarium (HUAA) [14]. A total of 2.5 kg of fresh plant material was collected and allowed to dry at room temperature, in total darkness, for 30 days.

### 4.2. Sample Preparation for Phytochemical Analysis and LC-MS/MS Conditions

The identification and quantification of phenolic acids and flavonoids were realized by high-performance liquid chromatography coupled with mass spectrometry using authenticated standards, as proposed by Vu y Alvarez [48]. Dried *L. virginicum* stems (50 mg) were mixed with 100% of methanol (900 μL), and homogenized using two stainless steel beads (SSB 32) in a TissueLyserII (Qiagen, Hilden, Germany) operated at 10 Hz for 5 min. The homogenized samples were centrifuged (16,000× *g*), and the supernatant was recovered. The extraction and centrifugation processes were repeated in the remaining pellet until the sample was clarified. The supernatants were vacuum-dried using a SAVANT speed-vac (Thermo Scientific^TM^, Waltham, MA, USA), and then used for chromatographic and mass spectrometric analysis, which was performed using a Shimadzu Nexera II HPLC system (Shimadzu Scientific Instruments Inc., Columbia, MD, USA) hyphenated with a Sciex QTRAP 6500+ mass spectrometer (AB Sciex Pte. Ltd., Framingham, MA, USA). Analyst software (version 1.6.3) was employed for sample acquisition and data analysis. 

The chromatographic system was equipped with a ZORBAX Eclipse XDB C18 column (2.1 mm × 100 mm, Agilent, Santa Clara, CA, USA). The separation process was performed in an elution gradient composed of a mixture of 2% acetic acid (A) and 100% acetonitrile (B) (0–1 min, 6% B, 1–5 min, 17% B, 5–8 min, 20% B, 8–16 min, 90% B, 16–18 min, hold at 90% B, 18–19 min, 6% B) with a flow rate of 0.4 mL/min. In our investigation, the authenticated standards employed were apigenin, apigeninidin, caffeic acid, catechin, naringenin chalcone, chlorogenic acid, cinnamic acid, cyanidin, daidzein, delphinidin, epicatechin, ferulic acid, gallic acid, genistein, hesperetin, kaempferol, luteolin, naringenin, p-coumaric acid, phloretin, proanthocyanidin A2, procyanidin B2, protocatechuic acid, quercetin, quercetin-3-galactoside, quercetin-3-glucoside, resveratrol, rutin, syringic acid, and vanillic acid.

### 4.3. Preparation of ELv for Biological Assay

The dry plants of *L. virginicum* were separated into stems, leaves and seeds. The stems were chosen for their higher phenolic and flavonoid content compared to the leaves and roots, as reported by Cruz et al. [14]. The dried stems were pulverized using a mechanical grinder. Then, 2.5 kg of powder was subjected to maceration with methanol (99.8%, JT Baker^®^, Phillipsburg, NJ, USA) in an Erlenmeyer flask at room temperature for 24 h. The macerate was filtered through Whatman qualitative filter paper No. 1 and concentrated using a rotary evaporator (Sev-Prendo^®^, Puebla, Mexico) operated at 40 °C. The obtained methanolic extract (ELv, 25.7 g) was stored at −4 °C in dark conditions. Stock solutions of extract were prepared fresh. An aliquot of 100 mg of dried methanolic extract was resuspended in 10 mL ethanol (20%). Serial dilution of the stock solution with the appropriate volumes of DMEM medium afforded a final assay solution of 0.001, 0.01, 0.1 and 1 mg/mL as required. The final ethanol concentration in both test and vehicle solutions was 0.2%.

### 4.4. Evaluation of Metabolic Activity by MTT Assay 

Cell viability of Caco- 2 cells (ATCC^®^ HTB-37™) in the presence of the ELv was assessed by 3-(4,5-dimethylthiazol-2-yl)2,5-diphenyltetrazolium bromide (MTT) assay (Catalog No: M2128; Sigma-Aldrich, Toluca, Mexico), a sensitive and reliable colorimetric indicator of cellular metabolic activity. The human normal fibroblast cell line, Detroit 548 (ATCC^®^ CCL-116™), was used to determine the cancer-cell specificity of ELv. Both cell lines were seeded in a 96-well plate at a density of 1.5 × 10^5^ cells/well in DMEM/F-12 HAM medium supplemented with 10% fetal bovine serum and 1 µL/mL of penicillin/streptomycin antibiotic (100 IU/mL-100 µg/mL) and maintained in a 5% CO_2_ humidified incubator at 37 °C overnight. Subsequently, cells were treated with different concentrations of the ELv (0.001, 0.01, 0.1 and 1 mg/mL) by diluting the ELv in cell media and incubated at 37 °C in a humid atmosphere with 5% CO_2_ for 24 or 48 h. Cells treated with 2% Triton X-100 were used as a positive control of cell death to compare the level of cytotoxicity of the methanolic extract of *L. virginicum* because it has been shown that Triton X-100 induces apoptotic and necrotic death in prostate and colon cancer cell lines [49]. After incubation, 100 μL of MTT solution (0.5 mg/mL) was added into each well and incubated at 37 °C in a humid atmosphere with 5% CO_2_ for 4 h. Following this, the medium was carefully aspirated and replaced with 200 µL of 0.1 N acid isopropanol to solubilize the formazan crystals. A plate reader (Multiskan Sky, Thermo Scientific™ Waltham, MA, USA) was used to quantify the solubilized formazan at a wavelength of 595 nm, where higher absorbance was proportional to metabolically active (thus living) cells and vice versa. The relative cell viability rate (%) was calculated using a formula: (absorbance treated cells − absorbance blank cells)/(absorbance control cells − absorbance blank cells) × 100%. Data are presented as the average of three to six independent experiments carried out in triplicate, and the half-maximal lethal concentration (IC50) was calculated by GraphPad Prism 8 with a logistic function. 

### 4.5. Lactate Dehydrogenase (LDH) Release Assay

The cytotoxic effect of the ELv on Caco-2 and Detroit 548 cells was evaluated by measuring LDH release using the Cytotoxicity Detection kit (Catalog No: 11644793001; Roche, Mannheim, Germany). Both cells were cultured in a 96-well plate at a density of 3.0 × 10^4^ cells/well in DMEM/F12 medium for 24 h to allow cell adherence. The cells were then treated with different concentrations of the ELv (0.01–1 mg/mL) for 24 and 48 h at 37 °C in a 5% CO_2_ humid incubator. After this period, 100 μL of supernatant or cell lysate was transferred to a new 96-well plate. Then, 100μL of LDH reaction mixture was added to each well according to the kit manufacturer’s specifications. To determine the control absorbance value, 100 μL DMEM/F12 was added to the plate plus 100 μL LDH reaction mixture (control). As a positive control, the cells were exposed to a lysis solution (2% triton X-100 in DMEM medium), and 100 μL LDH reaction mixture was added to determine the maximal absorbance. All samples were incubated at 37 °C for 30 min in darkness. The absorbance was quantified in a Multiskan Sky plate reader (Thermo Scientific^®^) at a wavelength of 492 nm. We performed three independent experiments in triplicate. The results are expressed in % LDH release in relation to the control.

### 4.6. DNA Fragmentation Analysis

The Cellular DNA Fragmentation ELISA kit (Catalog. No. 11585045001; Roche Applied Science, Mannheim, Germany) was used according to the manufacturer’s instructions. Caco-2 cells were seeded at a density of 1.5 × 10^5^ cells/well in 96-well plates in DMEM/F12 medium for 24 h. Then, cells were incubated for 48 h with different concentrations of the ELv (0.01, 0.1 and 1 mg/mL). Subsequently, 10 μM BrdU was added and allowed to react for 24 h. The supernatant was then collected and transferred to a 96-well plate previously coated with the anti-BrdU antibody. As a negative control, BrdU-untreated cells were lysed with the incubation buffer solution, and the lysate was placed on the antibody-coated plate. BrdU-labeled DNA fragments were detected from damaged cells using the plate reader (Multiskan Sky; Thermo Scientific™, Waltham, MA, USA) at a wavelength of 370 nm. Three independent experiments were performed in triplicate.

### 4.7. RNA Isolation and Real-Time PCR

Total RNA was extracted from Caco-2 cells treated with 1 mg/mL of the *L. virginicum* methanolic extract for 48 h using the TRIzol-Chloroform method (TRI reagent^®^; Catalog No: T9424; Sigma-Aldrich, Toluca, México) according to the manufacturer’s protocol. The RNA obtained was quantified with a BioDrop™µLITE (Harvard Bioscience, Inc.; Holliston, MA, USA) and its quality was calculated based on the A260/A280 and A260/A230 nm absorbance ratios; the accepted ratio range was 1.9–2.0. One µg of total RNA was used for cDNA synthesis using First Strand cDNA Synthesis Kit (Catalog No: K1612; Thermo Fisher Scientific™, Waltham, MA, USA) with the oligo(dT)18 in a T100 Thermal Cycler (Bio-Rad; Hercules, CA, USA) according to the manufacturer’s indications. Once the cDNA was obtained, the gene expression of p53, Bcl-2, and Caspase 3 was quantitatively determined by CFX96TM Real-Time PCR (Bio-Rad) using SsoAdvanced™ Universal SYBR^®^ Green Master Supermix Kit (Catalog No: 172-5270; Bio-Rad; Hercules, CA, USA) according to the manufacturer’s protocol. Primer sequences used are listed in Table 3. Expression levels were determined in three independent experiments in triplicate with the 2^−ΔΔCt^ method using glyceraldehyde 3-phosphate dehydrogenase (GAPDH) as a constitutive expression control transcript and represented as fold change.

### 4.8. Statistical Analysis

Statistical analyses were performed in GraphPad Prism version 8 (GraphPad Software, La Jolla, CA, USA). Data values are presented as the mean ± standard error of the mean (SEM). Cells from at least three different experiments in triplicate were used for each experimental protocol. Ordinary two-way ANOVA with Dunnett’s multiple comparison test was used to determine the statistical differences between the groups for all parameters, unless otherwise mentioned. Differences with a value of *p* ≤ 0.05 were considered statistically significant.

## Figures and Tables

**Table 1:** Detection and quantification of flavonoids (ng/g) in the *L. virginicum* stem.

Flavonoids	Concentration (ng/g)
Quercetin-3-galactoside	1965.71
Quercetin-3-glucoside	379.48
Kaempferol	86.22
Quercetin	52.31
Naringenin + Naringenin chalcone	4.93

**Table 2:** Detection and quantification of phenolic acids (ng/g) in the *L. virginicum* stem.

Phenolic Acids	Concentration (ng/g)
Cinnamic acid	3466.98
*p*-Coumaric acid	2299.60
Ferulic acid	1215.49
Vanillic acid	941.68
Caffeic acid	629.79
Chlorogenic acid	297.63
Syringic acid	292.42
Gallic acid	6.37

**Table 3:** Primer sequences used in real-time PCR assay in Caco-2 cell line.

Gene Accession Number	Sequence (5′-3′)	bp	Annealing Tm (°C)
Caspase 3 (NM_032991)	Fw: CAATGGACTCTGGATATCC Rv: GCTGCATCGACATCTGTAC	139	55
Bcl-2 (NM_000633.3)	Fw: GACTGAGTACCTGAACCGGC Rv: GCAGAGTCTTCAGAGACAGC	131	55
p53 (NM_001407269.1)	Fw: CGACATAGTGTGGTGGTGCC Rv: CCATGCAGGAACTGTTACAC	94	55
GAPDH (NM 001289726.1)	Fw: AGTCTACTGGCGTCTTCACC Rv: CCACGATGCCAAAGTTGTCA	225	60

Fw: Forward; Rv: reverse; GAPDH: Glyceraldehyde-3-phosphatase dehydrogenase; bp: expected product length in base pairs and Tm: temperature.

## Data Availability

The original contributions presented in the study are included in the article, further inquiries can be directed to the corresponding authors.

## References

[1] Sung H., Ferlay J., Siegel R.L., Laversanne M., Soerjomataram I., Jemal A., Bray F. (2021). Global cancer statistics 2020: GLOBOCAN estimates of incidence and mortality worldwide for 36 cancers in 185 countries. CA Cancer J. Clin..

[2] Ciardiello F., Ciardiello D., Martini G., Napolitano S., Tabernero J., Cervantes A. (2022). Clinical management of metastatic colorectal cancer in the era of precision medicine. CA Cancer J. Clin..

[3] Riedesser J.E., Ebert M.P., Betge J. (2022). Precision Medicine for Metastatic Colorectal Cancer in Clinical Practice. Ther. Adv. Med. Oncol..

[4] Modest D.P., Pant S., Sartore-Bianchi A. (2019). Treatment sequencing in metastatic colorectal cancer. Eur. J. Cancer.

[5] Emran T.B., Shahriar A., Mahmud A.R., Rahman T., Abir M.H., Siddiquee M.F., Ahmed H., Rahman N., Nainu F., Wahyudin E. (2022). Multidrug resistance in cancer: Understanding molecular mechanisms, immunoprevention and therapeutic approaches. Front. Oncol..

[6] Ocran Mattila P., Ahmad R., Hasan S.S., Babar Z.U. (2021). Availability, affordability, access, and pricing of anti-cancer medicines in low- and middle-income countries: A systematic review of literature. Front. Public Health.

[7] Dehelean C.A., Marcovici I., Soica C., Mioc M., Coricovac D., Iurciuc S., Cretu O.M., Pinzaru I. (2021). Plant-derived anti-cancer compounds as new perspectives in drug discovery and alternative therapy. Molecules.

[8] Peña M., Guzmán A., Martínez R., Mesas C., Prados J., Porres J.M., Melguizo C. (2022). Preventive effects of *Brassicaceae* family for colon cancer prevention: A focus on in vitro studies. Biomed. Pharmacother..

[9] Ryan G. (1998). Medical ethnobiology of the highland Maya of Chiapas, Mexico: The gastrointestinal diseases. Am. J. Hum. Biol..

[10] Calzada F., Barbosa E., Cedillo-Rivera R. (2003). Antiamoebic activity of benzyl glucosinolate from *Lepidium virginicum*. Phytother. Res..

[11] Osuna L., Tapia-Pérez M.E., Figueroa O., Jiménez-Ferrer E., Garduño-Ramírez M.L., González-Garza M.T., Carranza-Rosales P., Cruz-Vega D.E. (2006). Micropropagation of *Lepidium virginicum* (*Brassicaceae*), a plant with antiprotozoal activity. In Vitro Cell. Dev. Biol. -Plant.

[12] Pacheco-Hernández Y., Santamaría-Juárez J.D., Hernández-Silva N., Cruz-Durán R., Mosso-González C., Villa-Ruano N. (2021). Essential oil of *Lepidium virginicum*: Protective activity on anthracnose disease and preservation effect on the nutraceutical content of tamarillo fruit (*Solanum betaceum*). Chem. Biodivers..

[13] Chae Y.H., Shin D.Y., Park C., Lee Y.T., Moon S.G., Choi Y.H. (2011). Induction of apoptosis in human colon carcinoma HCT116 cells using a water extract of *Lepidium virginicum* L.. J. Korean Soc. Food Sci. Nutr..

[14] Cruz-Muñoz J.R., Barrios-García T., Valdez-Morales E.E., Durán-Vazquez M.F., Méndez-Rodríguez K.B., Barajas-Espinosa A., Ochoa-Cortes F., Martínez-Saldaña M.C., Gómez-Aguirre Y.A., Alba R.G. (2022). Ethanolic extract from *Lepidium virginicum* L. ameliorates DNBS-induced colitis in rats. J. Ethnopharmacol..

[15] Rana A., Samtiya M., Dhewa T., Mishra V., Aluko R.E. (2022). Health benefits of polyphenols: A concise review. J. Food Biochem..

[16] Martín Gordo D.A. (2018). Los compuestos fenólicos, un acercamiento a su biosíntesis, síntesis y actividad biológica. RIAA.

[17] Yábar E., Chirinos R., Campos D. (2019). Compuestos fenólicos y capacidad antioxidante en tres ecotipos de Maca (*Lepidium meyenii* Walp.) durante la pre-cosecha, cosecha y secado natural post-cosecha. Sci. Agropecu..

[18] Ohishi T., Kaneko M.K., Yoshida Y., Takashima A., Kato Y., Kawada M. (2023). Current targeted therapy for metastatic colorectal cancer. Int. J. Mol. Sci..

[19] Rumpold H., Niedersüß-Beke D., Heiler C., Falch D., Wundsam H.V., Metz-Gercek S., Piringer G., Thaler J. (2020). Prediction of mortality in metastatic colorectal cancer in a real-life population: A multicenter explorative analysis. BMC Cancer.

[20] Shen L., Li Q., Wang W., Zhu L., Zhao Q., Nie Y., Zhu B., Ma D., Lin X., Cai X. (2020). Treatment patterns and direct medical costs of metastatic colorectal cancer patients: A retrospective study of electronic medical records from urban China. J. Med. Econ..

[21] Sougklakos I., Athanasiadis E., Boukovinas I., Karamouzis M., Koutras A., Papakotoulas P., Latsou D., Hatzikou M., Stamuli E., Balasopoulos A. (2020). Treatment pathways and associated costs of metastatic colorectal cancer in Greece. cost effectiveness and resource allocation. Cost Eff. Resour. Alloc..

[22] Rayan A., Raiyn J., Falah M. (2017). Nature is the best source of anti-cancer drugs: Indexing natural products for their anti-cancer bioactivity. PLoS ONE.

[23] Çetinkaya S., Çınar Ayan İ., Süntar İ., Dursun H.G. (2022). The phytochemical profile and biological activity of liquidambar orientalis Mill. var. orientalis via NF-κB and Apoptotic Pathways in Human Colorectal Cancer. Nutr. Cancer.

[24] Islam B.U., Suhail M., Khan M.K., Zughaibi T.A., Alserihi R.F., Zaidi S.K., Tabrez S. (2021). Polyphenols as anti-cancer agents: Toxicological concern to healthy cells. Phytother. Res..

[25] Almatroodi S.A., Alsahli M.A., Almatroudi A., Verma A.K., Aloliqi A., Allemailem K.S., Khan A.A., Rahmani A.H. (2021). Potential therapeutic targets of quercetin, a plant flavonol, and its role in the therapy of various types of cancer through the modulation of various cell signaling pathways. Molecules.

[26] Shabir I., Kumar Pandey V., Shams R., Dar A.H., Dash K.K., Khan S.A., Bashir I., Jeevarathinam G., Rusu A.V., Esatbeyoglu T. (2022). Promising bioactive properties of quercetin for potential food applications and health benefits: A review. Front. Nutr..

[27] Nile A., Nile S.H., Shin J., Park G., Oh J.W. (2021). Quercetin-3-glucoside extracted from apple pomace induces cell cycle arrest and apoptosis by increasing intracellular ROS levels. Int. J. Mol. Sci..

[28] Ackland M.L., van de Waarsenburg S., Jones R. (2005). Synergistic antiproliferative action of the flavonols quercetin and kaempferol in cultured human cancer cell lines. In Vivo.

[29] Zhao Z., Jin G., Ge Y., Guo Z. (2019). Naringenin inhibits migration of breast cancer cells via inflammatory and apoptosis cell signaling pathways. Inflammopharmacology.

[30] Arul D., Subramanian P. (2013). Naringenin (citrus flavonone) induces growth inhibition, cell cycle arrest and apoptosis in human hepatocellular carcinoma cells. Pathol. Oncol. Res..

[31] Lim W., Park S., Bazer F.W., Song G. (2017). Naringenin-induced apoptotic cell death in prostate cancer cells is mediated via the PI3K/AKT and MAPK signaling pathways. J. Cell. Biochem..

[32] Park J.H., Jin C.Y., Lee B.K., Kim G.Y., Choi Y.H., Jeong Y.K. (2008). Naringenin induces apoptosis through downregulation of Akt and caspase-3 activation in human leukemia THP-1 cells. Food Chem. Toxicol..

[33] Rosa L.D.S., Jordão N.A., Da Costa Pereira Soares N., DeMesquita J.F., Monteiro M., Teodoro A.J. (2018). Pharmacokinetic, antiproliferative and apoptotic effects of phenolic acids in human colon adenocarcinoma cells using in vitro and in silico approaches. Molecules.

[34] Shrihastini V., Muthuramalingam P., Adarshan S., Sujitha M., Chen J.T., Shin H., Ramesh M. (2021). Plant derived bioactive compounds, their anti-cancer effects and in silico approaches as an alternative target treatment strategy for breast cancer: An updated overview. Cancers.

[35] Kar N., Gupta D., Bellare J. (2021). Ethanol affects fibroblast behavior differentially at low and high doses: A comprehensive, dose-response evaluation. Toxicol. Rep..

[36] Özdemir A., Yildiz M., Senol F.S., Şimay Y.D., Ibişoglu B., Gokbulut A., Orhan I.E., Ark M. (2017). Promising anticancer activity of *Cyclotrichium niveum* L. extracts through induction of both apoptosis and necrosis. Food Chem. Toxicol..

[37] Laila F., Fardiaz D., Yuliana N.D., Damanik M.R.M., Nur Annisa Dewi F. (2020). Methanol extract of *Coleus amboinicus* (Lour) exhibited antiproliferative activity and induced programmed cell death in colon cancer cell WiDr. Int. J. Food Sci..

[38] Wong T.L., Strandberg K.R., Croley C.R., Fraser S.E., Nagulapalli Venkata K.C., Fimognari C., Sethi G., Bishayee A. (2021). Pomegranate bioactive constituents target multiple oncogenic and oncosuppressive signaling for cancer prevention and intervention. Semin. Cancer Biol..

[39] Srivastavaa N., Saxena A.K. (2023). Caspase-3 activators as anti-cancer agents. Curr. Protein Pept. Sci..

[40] Kashaw S.K., Agarwal S., Mishra M., Sau S., Iyer A.K. (2019). Molecular docking analysis of caspase-3 activators as potential anti-cancer agents. Curr. Comput. Aided Drug Des..

[41] Ozaki T., Nakagawara A. (2011). Role of p53 in cell death and human cancers. Cancers.

[42] Aubrey B.J., Kelly G.L., Janic A., Herold M.J., Strasser A. (2018). How does p53 induce apoptosis and how does this relate to p53-mediated tumour suppression?. Cell Death Differ..

[43] Li X.L., Zhou J., Chen Z.R., Chng W.J. (2015). P53 mutations in colorectal cancer—Molecular pathogenesis and pharmacological reactivation. World J. Gastroenterol..

[44] Jan R., Chaudhry G.E. (2019). Understanding apoptosis and apoptotic pathways targeted cancer therapeutics. Adv. Pharm. Bull..

[45] Moldoveanu T. (2023). Apoptotic mitochondrial poration by a growing list of pore-forming BCL-2 family proteins. BioEssays.

[46] Jiang M., Milner J. (2003). Bcl-2 constitutively suppresses p53-dependent apoptosis in colorectal cancer cells. Genes Dev..

[47] Shehata M.G., Abu-Serie M.M., Abd El-Aziz N.M., El-Sohaimy S.A. (2021). Nutritional, phytochemical, and in vitro anti-cancer potential of sugar apple (*Annona squamosa*) fruits. Sci. Rep..

[48] Vu D.C., Alvarez S. (2021). Phenolic, carotenoid and saccharide compositions of vietnamese *Camellia sinensis* teas and herbal teas. Molecules.

[49] Borner M.M., Schneider E., Pirnia F., Sartor O., Trepel J.B., Myers C.E. (1994). The detergent Triton X-100 induces a death pattern in human carcinoma cell lines that resembles cytotoxic lymphocyte-induced apoptosis. FEBS Lett..

